# Supplementary material for: GFI1 facilitates efficient DNA repair by regulating PRMT1 dependent methylation of MRE11 and 53BP1
Source: Nat Commun. 2018 Apr 12;9:1418. doi: 10.1038/s41467-018-03817-5 (PMC5897347; doi:10.1038/s41467-018-03817-5)
Supplement: Supplementary file 1 — Supplementary Information [file 41467_2018_3817_MOESM1_ESM.pdf]

GFI1 facilitates efficient DNA Repair by regulating PRMT1 dependent methylation of MRE11 and 53BP1

Vadnais et al.

Supplementary Information

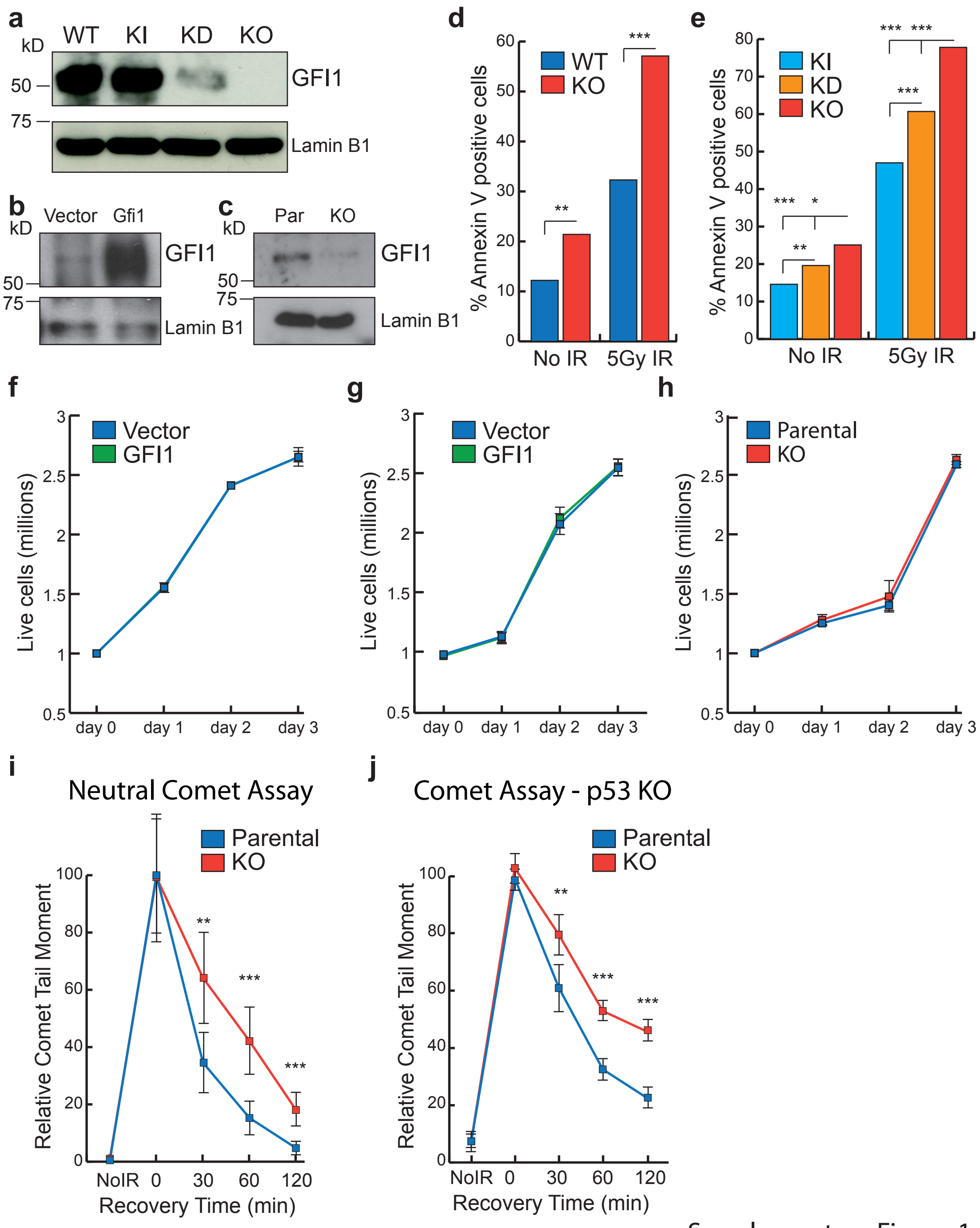

Supplementary Figure 1

Supplementary Figure 1
a Western blot analysis of GFI1 levels in thymocytes extracted from *Gfi1* WT, KI, KD, KO.
b Western blot analysis of GFI1 levels in SupT1 cells overexpressing GFI1 and control cells.
c Western blot analysis of GFI1 levels in parental and CRISPR-KO Jurkat cells.
d Thymocytes were extracted from *Gfi1* WT and age and sex matched *Gfi1* KO mice, exposed to 5 Gy IR, stained for Annexin V and 50,000 cells were analyzed by FACS 4 hours following exposure. One of 3 replicate experiments is shown. * = p <0.05, ** = p<0,01, *** = p<0,001 on a Fisher's test.
e Thymocytes from *Gfi1* KI, KD and KO mice were treated as in **a**.
f GFI1 overexpressing SupT1 cells and vector control cells were seeded at 1 million cells per ml and left untreated as controls for the irradiated cells in **Figure 1 a**. Cells were counted each following day. Error bars represent s.d.
g GFI1 overexpressing SupT1 cells and vector control cells were seeded at 1 million cells per ml and left untreated as controls for the Cytarabine treated cells in **Figure 1 b**. Cells were counted each following day.
h Jurkat cells with GFI1 KO and parental control cells were seeded at 1 million cells per ml and left untreated as controls for the cells in **Figure 1 c**. Cells were counted each following day.
i Jurkat cells with GFI1 KO and parental control cells were exposed to 5 Gy IR and allowed to recover for the indicated time. Cells were then lysed and analyzed by neutral Comet Assay. Comet tail moment averages are shown. One of 3 replicate experiments is shown. Error bars represent s.d.
j Thymocytes were extracted from *Gfi1* WT and age and sex matched *Gfi1* KO mice, exposed to 5 Gy IR and allowed to recover for the indicated time. Cells were then lysed and analyzed by alkaline Comet Assay. Comet tail moment averages are shown. One of 3 replicate experiments is shown. Error bars represent s.d.

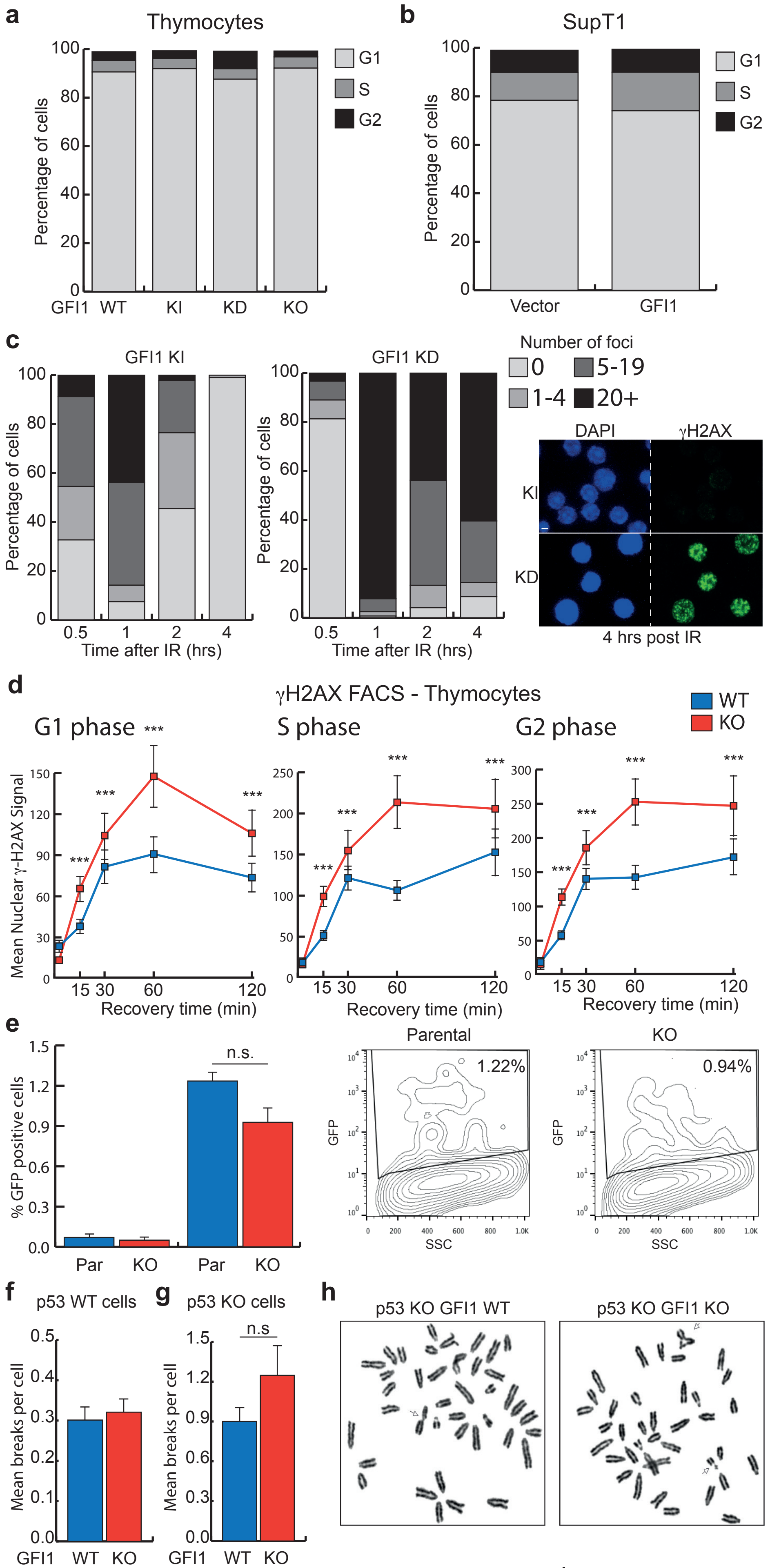

Supplementary Figure 2

Supplementary Figure 2

a Thymocytes extracted from *Gfi1* WT and age and sex matched KI, KD and KO mice were fixed in paraformaldehyde, stained with Propidium Iodide and measured by FACS for cell cycle distribution.

b GFI1 overexpressing SupT1 cells and vector control cells were treated as in **a** and analyzed for cell cycle distribution.

c Thymocytes extracted from *Gfi1* KI and matching KD mice were exposed to 5 Gy IR and allowed to recover for the indicated time. Cells were spread on glass slides, fixed and stained for γ-H2AX. Percentages of cells displaying the indicated number of γ-H2AX foci are represented in graph. Representative images of the 4 hour time-point. Scale bar represents 10μm.

d Thymocytes extracted from *Gfi1* WT and age and sex matched *Gfi1* KO mice were exposed to 5 Gy IR and allowed to recover for the indicated time. Cells were stained for γ-H2AX and PI and analyzed by FACS. Mean γ-H2AX signal and standard deviation are shown.

e Left: GFI1 KO Jurkat T cells and parental control cells were electroporated with the EJ5-GFP plasmid and with a plasmid encoding the I-SceI restriction enzyme. GFP signal was measured by FACS 72 hours after electroporation. Cells electroporated without I-SceI plasmid are shown as control. Results from one representative triplicate experiment are shown. Right: Representative FACS profile showing GFP signal versus side scatter. Positive gate determined using cells electroporated without I-SceI plasmid.

f CD4+ T cells were extracted from *Gfi1* WT and age and sex matched KO mice and grown for 72 hours following activation with α-CD3 and α-CD28 antibodies. The mean number of breaks was counted in at least 50 metaphases from 3 mice of each genotype.

g Cells were extracted from *Gfi1* wt, p53 KO and age and sex matched *Gfi1* KO, p53 KO mice and breaks were quantified as in **e**.

h Representative images of chromosome breaks in T cell metaphases from **f**. Chromosome breaks are indicated with arrows.

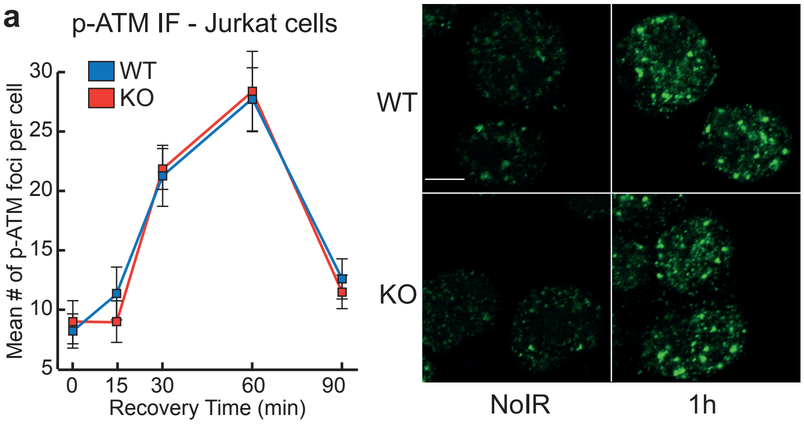

Supplementary Figure 3

Supplementary Figure 3

GFI1 KO and parental control Jurkat cells were exposed to 5Gy IR and allowed to recover for the indicated time. Cells were then spread on glass slides using a cytospin, fixed and stained for p−ATM. The mean numbers of p-ATM foci are are shown. One of 3 replicate experiments is shown. Error bars represent s.d. * = p <0.05, ** = p<0,01, *** = p<0,001 on a Welch corrected T test.

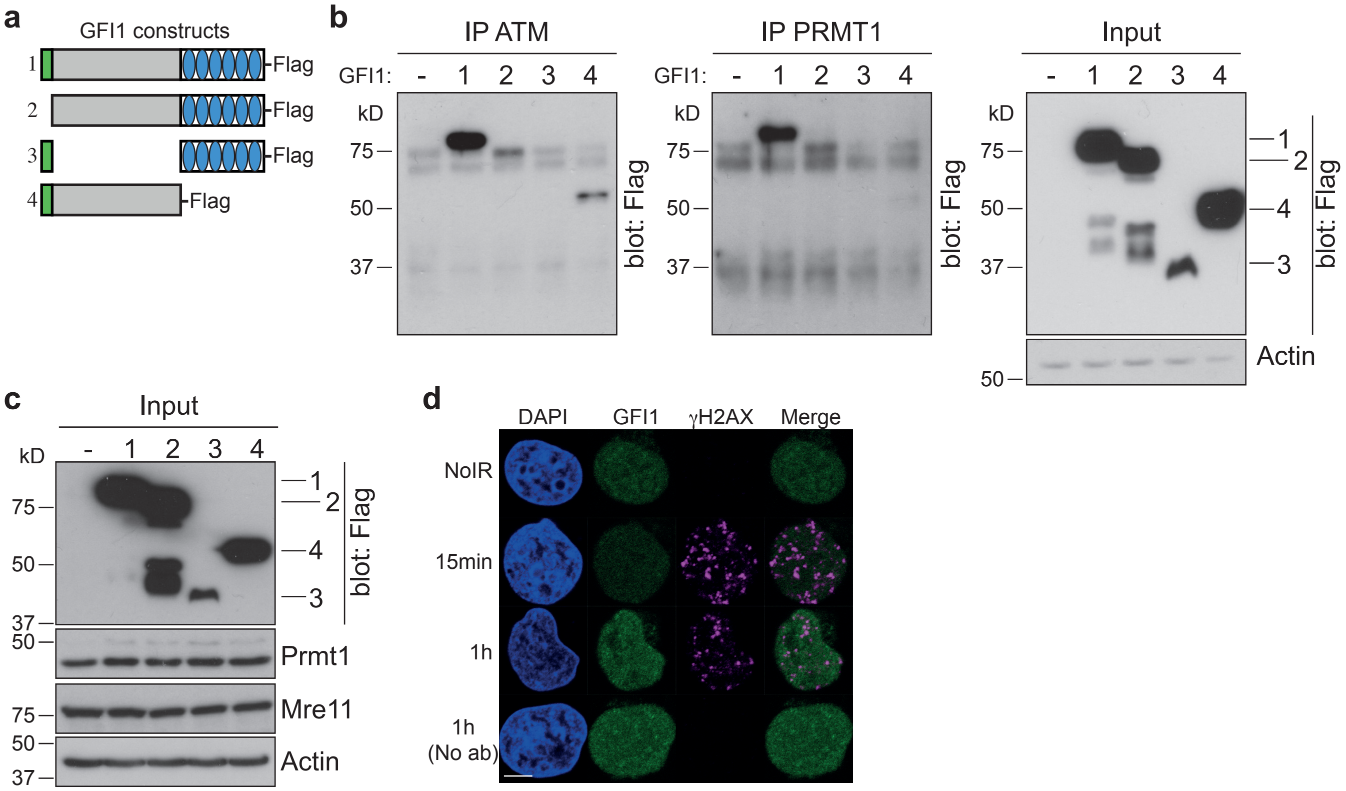

Supplementary Figure 4

Supplementary Figure 4

a Diagram of the different GFI1 fusion proteins used, as seen in figure 3 f.

b 293T cells expressing variants of the GFI1-Flag fusion protein seen in a were immunoprecipitated for the indicated protein. Extracts were separated by SDS-PAGE and blotted for Flag.

c Inputs from 293T cells as in b (see also figure 3 f-g) were blotted for the indicated proteins.

d SupT1 cells expressing a GFI1-GFP fusion protein were spread on glass slides using a Cytospin, stained for γ-H2AX and visualized for immunofluorescence by confocal microscopy. Control cells treated without primary antibody but with secondary antibodies are shown. Scale bar represents 10μm.

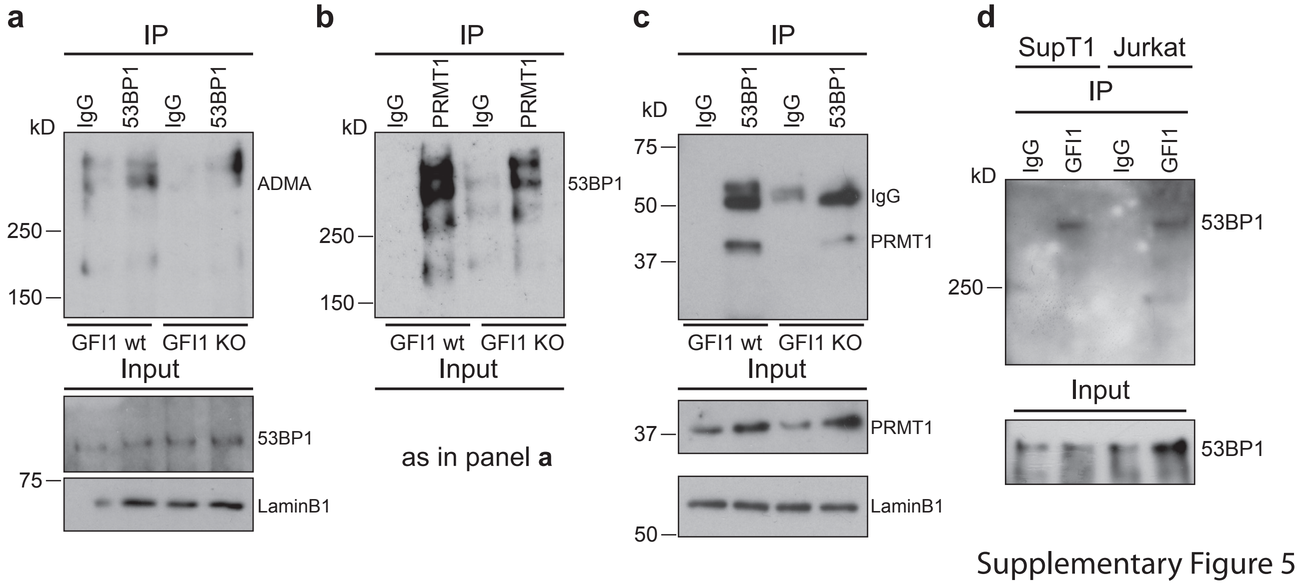

Supplementary Figure 5

a Nuclear extracts were prepared from thymocytes extracted from Gfi1 WT mice and matching Gfi1 KO mice and were immunoprecipitated for 53BP1 and blotted for ADMA.

b Extracts as in **b** were immunoprecipitated for PRMT1 and blotted for 53BP1. IP from panels b and c were prepared from the same cellular extracts and thus have the same loading controls.

c Extracts as in **b** were immunoprecipitated for 53BP1 and blotted for PRMT1.

d SupT1 and Jurakt cells were immunoprecipitated for GFI1 and extracts were blotted for 53BP1.

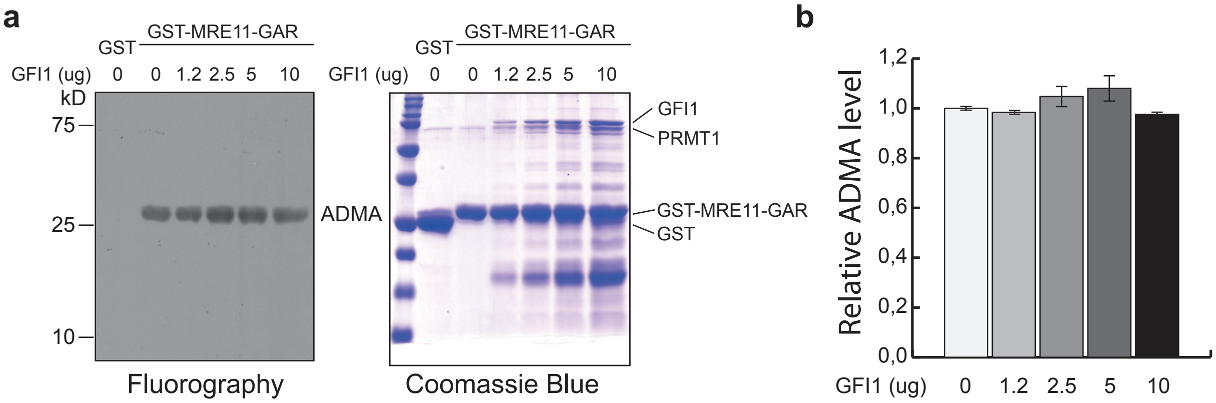

Supplementary Figure 6

Supplementary Figure 6

a Purified PRMT1 protein was incubated with GST-MRE11-GAR motif or GST control in the presence of radioactive Adenosyl-L-Methionine and increasing concentrations of purified GFI1 protein. Extracts were run on SDS-PAGE and exposed on film. Coomassie blue staining of gel is shown on the right.

b Quantification of normalized radioactive signal from 3 independent experiments carried out as in **a**.

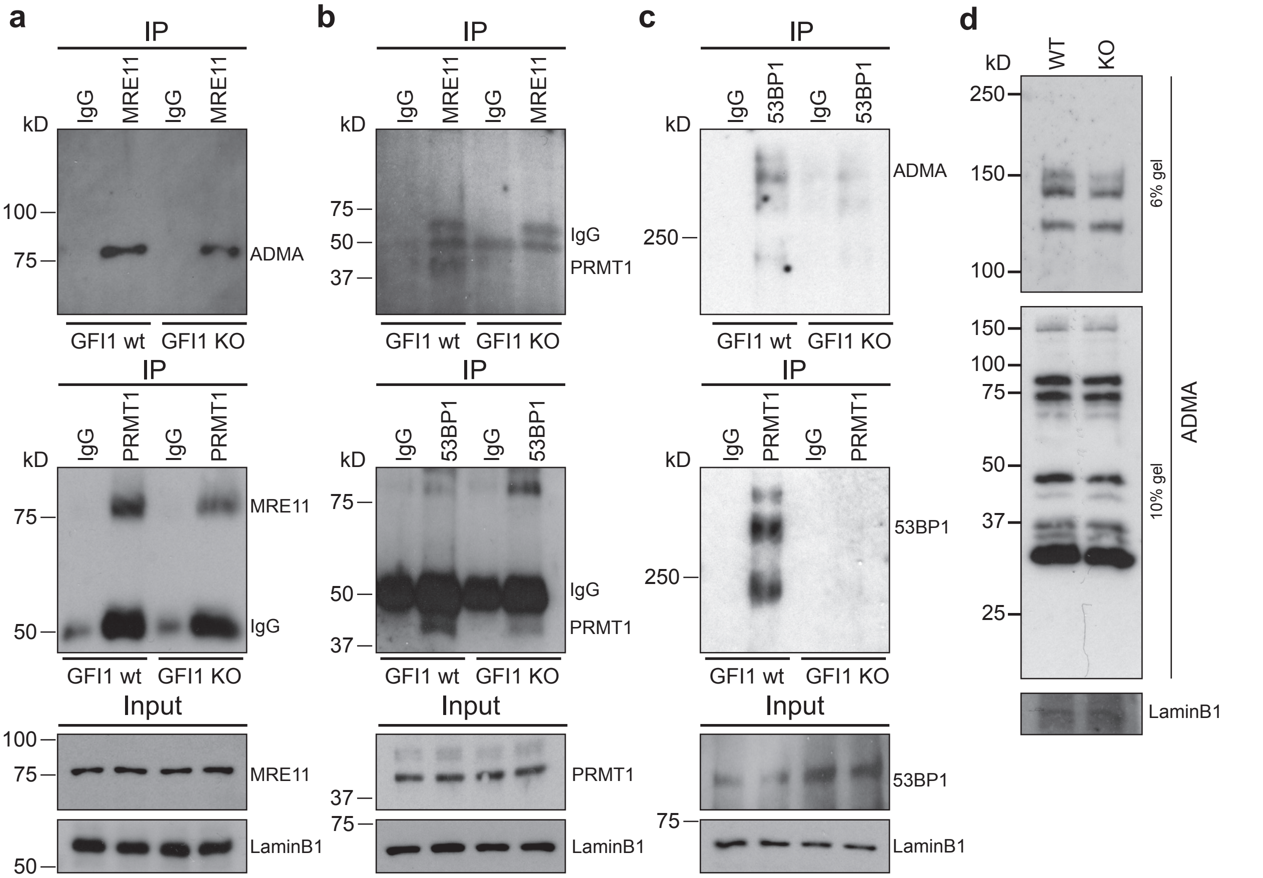

Supplementary Figure 7

Supplementary Figure 7

a Nuclear extracts were prepared from GFI1 KO Jurkat and parental control cells and were immunoprecipitated for MRE11 and PRMT1 and blotted for ADMA in the case of the MRE11 IP, and for MRE11 in the case of the PRMT1 IP.

b Extracts as in **a** were immunoprecipitated for MRE11 and 53BP1 and blotted for PRMT1.

c Extracts as in **a** were immunoprecipitated for 53BP1 and PRMT1 and blotted for ADMA in the case of the 53BP1 IP, and for 53BP1 in the case of the PRMT1 IP.

d Nuclear extracts were prepared from GFI1 KO Jurkat cells and parental control cells, were separated by SDS-PAGE and blotted for ADMA.

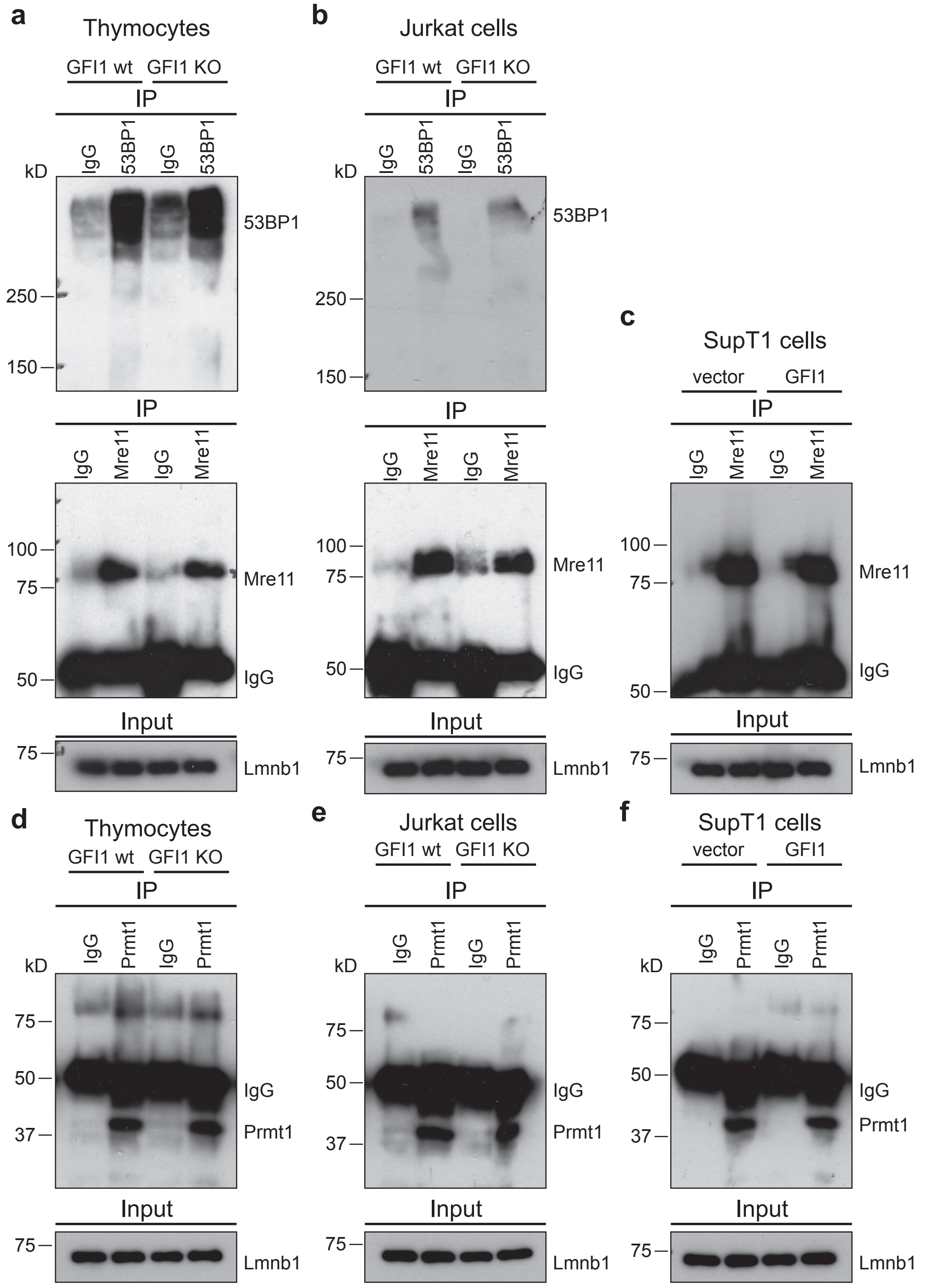

Supplementary Figure 8

Supplementary Figure 8

a Nuclear extracts prepared from thymocytes extracted from *Gfi1* WT mice and matching *Gfi1* KO mice were immunoprecipitated for 53BP1 and MRE11 and blotted for the same protein using a different antibody.

b Nuclear extracts prepared from GFI1 KO and parental control Jurkat cells were treated as in **a**.

c Nuclear extracts prepared from GFI1 overexpressing and vector control SupT1 cells were treated as in **a** but only for MRE11.

d Extracts as in **a** were immunoprecipitated for PRMT1 and blotted for the same protein using a different antibody.

e Extracts as in **b** were immunoprecipitated for PRMT1 and blotted for the same protein using a different antibody.

f Extracts as in **c** were immunoprecipitated for PRMT1 and blotted for the same protein using a different antibody.

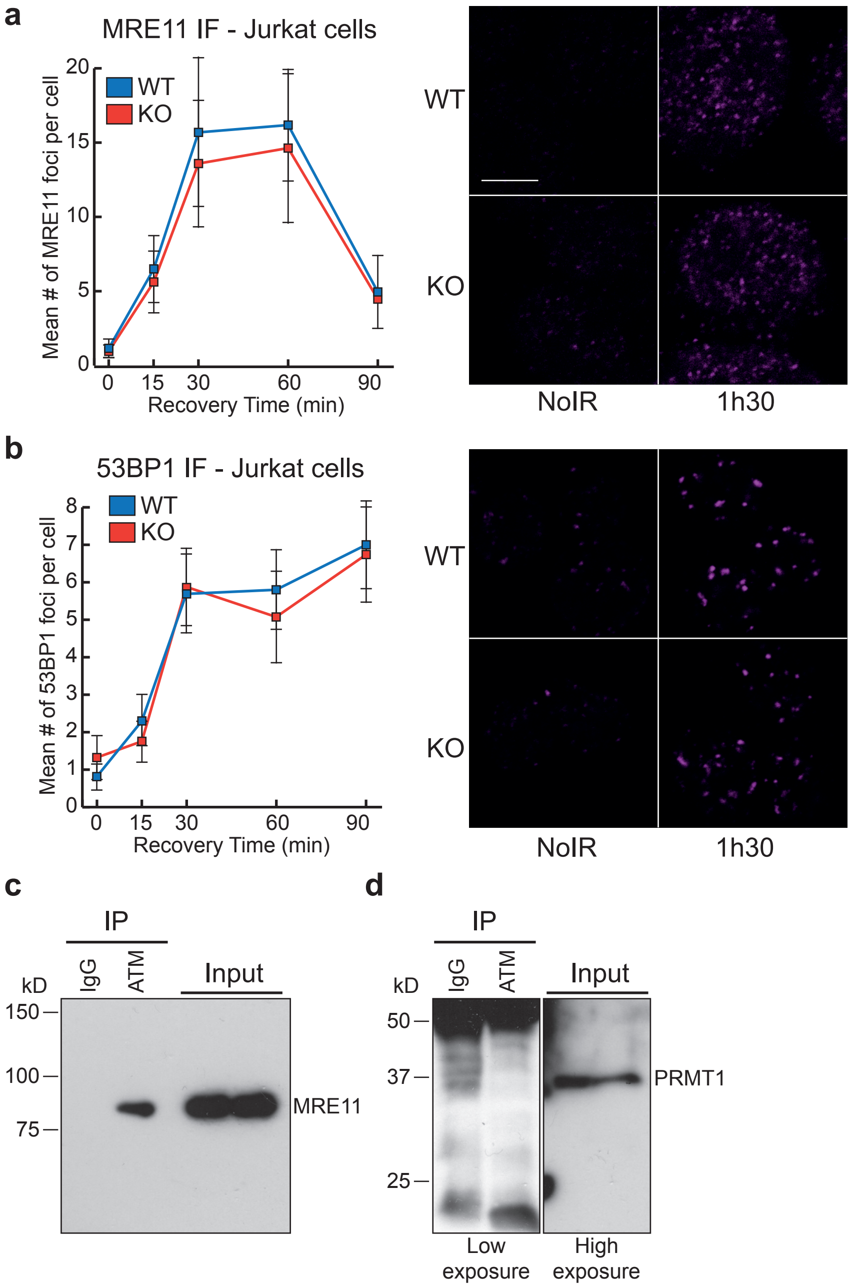

Supplementary Figure 9

Supplementary Figure 9

a GFI1 KO and parental control Jurkat cells were exposed to 5Gy IR and allowed to recover for the indicated time. Cells were then spread on glass slides using a cytospin, fixed and stained for MRE11. The mean numbers of MRE11 foci are shown. One of 3 replicate experiments is shown. Error bars represent s.d. * = p <0.05, ** = p<0,01, *** = p<0,001 on a Welch corrected T test. Scale bar represents 10μm.

b Cells treated as in **a** were stained for 53BP1. The mean numbers of 53BP1 foci are shown.

d Parental Jurkat cells were immunoprecipitated for ATM and nuclear extracts were blotted for MRE11. MRE11 signal in input samples is shown as a control.

e Parental Jurkat cells were immunoprecipitated for ATM and nuclear extracts were blotted for PRMT1. PRMT1 signal in input samples is shown as a control.

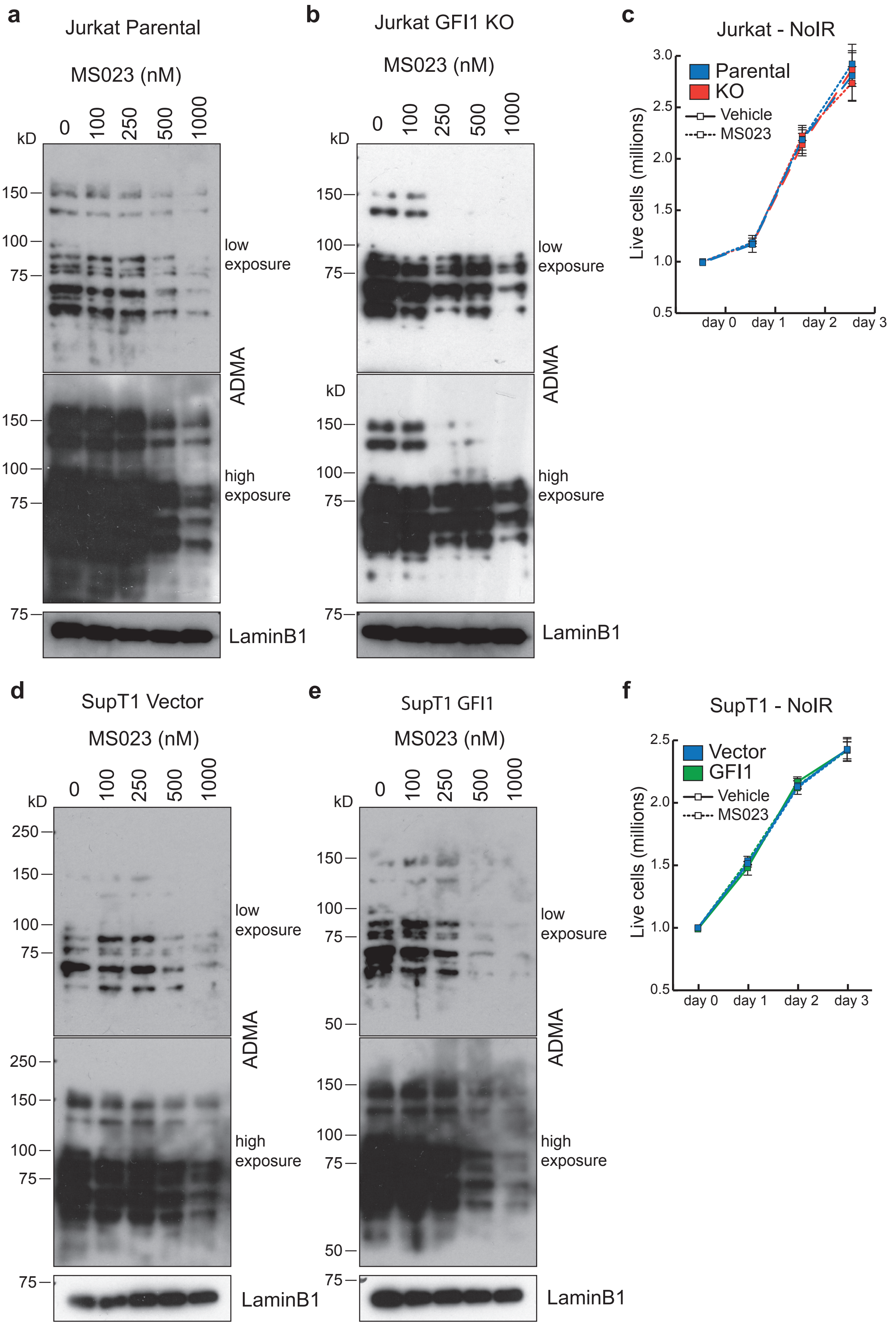

Supplementary Figure 10

Supplementary Figure 10
a Parental Jurkat cells were treated with the indicated concentrations of MS023. Whole nuclear extracts were separated by SDS-PAGE and blotted for ADMA.
b GFI1 KO Jurkat cells were treated and analyzed as in **a**.
c GFI1 KO Jurkat T cells and parental control cells treated with MS023 were seeded at 1 million cells per ml. Cells were counted each following day. Error bars represent s.d.
d SupT1 Vector control cells were treated and analyzed as in **a**.
e GFI1 overexpressing SupT1 cells were treated and analyzed as in **a**.
f SupT1 cells overexpressing GFI1 and vector control cells treated with MS023 were seeded at 1 million cells per ml. Cells were counted each following day.

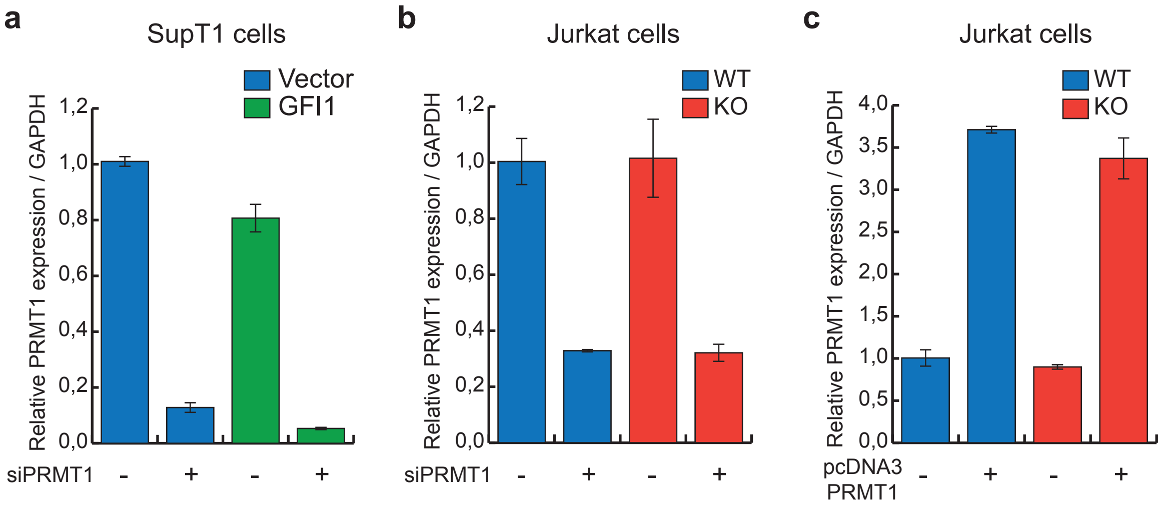

Supplementary Figure 11

Supplementary Figure 11

a cDNA was prepared from mRNA exptracted from GFI1 overexpressing and vector control SupT1 cells electroporated with PRMT1 siRNA or a non-targeting control siRNA (from Figure 6 **f**). PRMT1 mRNA levels were measured by qPCR using GAPDH as a control. Error bars represent s.d.

b cDNA was prepared from mRNA extracted from GFI1 KO and parental control Jurkat cells electroporated with PRMT1 siRNA or a non-targeting control siRNA (from Figure 6 **g**). PRMT1 mRNA levels were measured by qPCR using GAPDH as a control.

c cDNA was prepared from mRNA extracted from GFI1 KO and parental control Jurkat cells electroporated with a PRMT1 expression plasmid or an empty vector (from Figure 6 **h**). PRMT1 mRNA levels were measured by qPCR using GAPDH as a control.

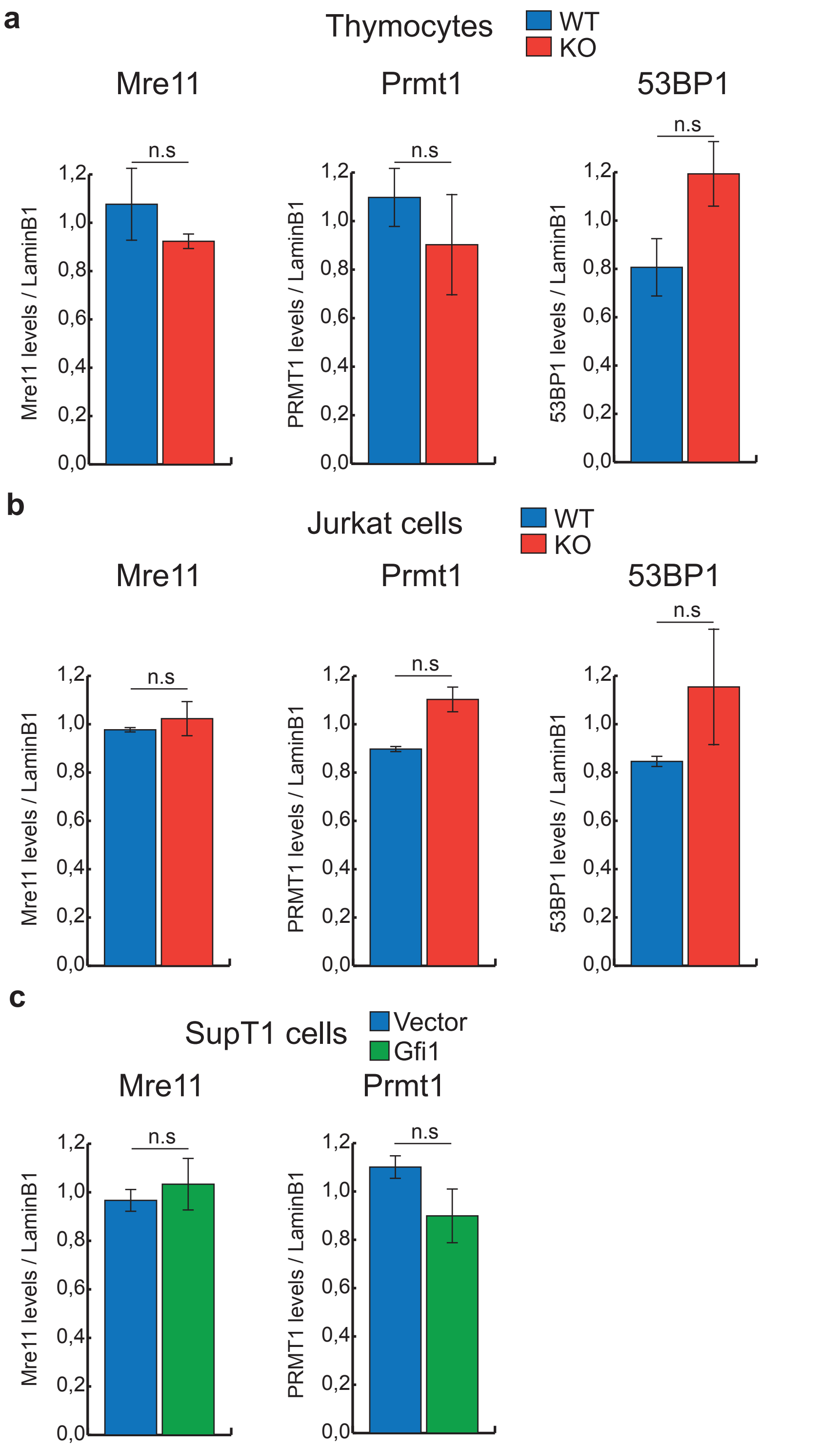

Supplementary Figure 12

Supplementary Figure 12

a Protein levels of the indicated protein were quantified from loading controls of thymocyte CoIP experiments in Figure 5 and Supplementary Figure 5 and normalized to their respective laminB1 loading controls. Average signal intensities were compared between GFI1 WT and KO thymocytes. "n.s." denotes no significant difference is found on a Welch corrected T test.

b Protein levels of the indicated protein were quantified from loading controls of Jurkat cells CoIP experiments in Supplementary Figure 7 and normalized to their respective laminB1 loading controls. Average signal intensities were compared between GFI1 WT and KO cells.

c Protein levels of the indicated protein were quantified from loading controls of SupT1 cells CoIP experiments in Supplementary Figure 5 and normalized to their respective laminB1 loading controls. Average signal intensities were compared between GFI1 overexpressing and vector control cells.

Figure 3d

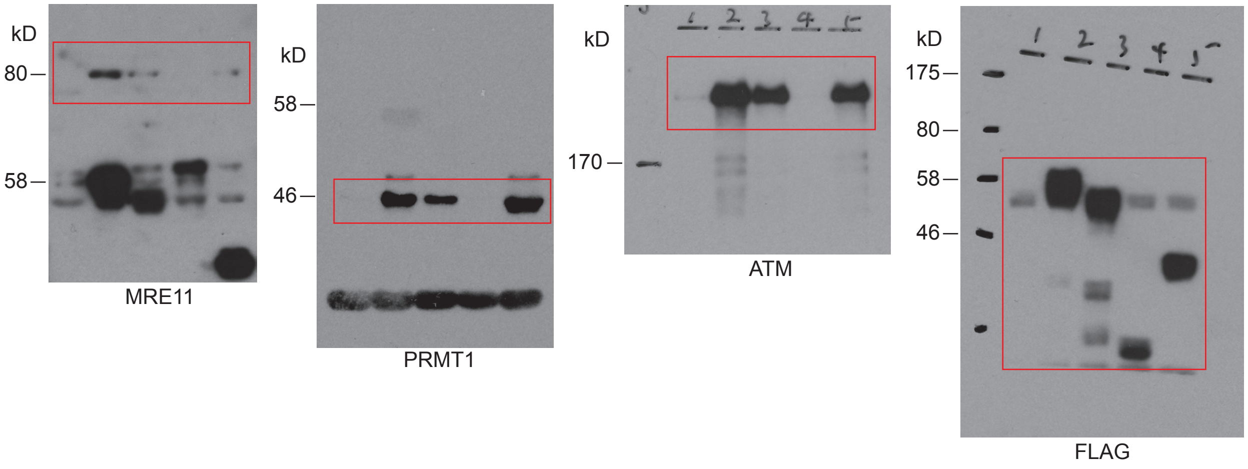

Figure 3e

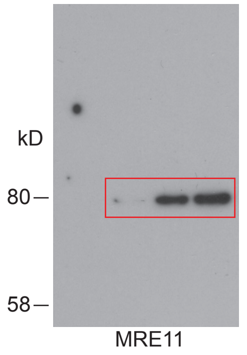

Figure 3h & i - MRE11

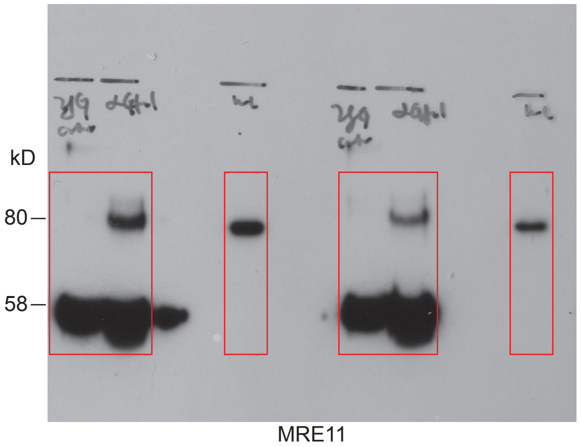

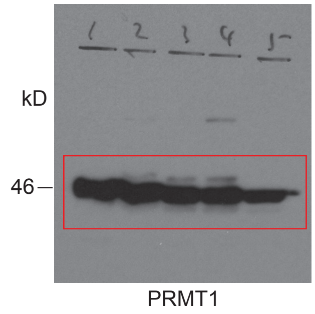

Figure 3h & i - PRMT1

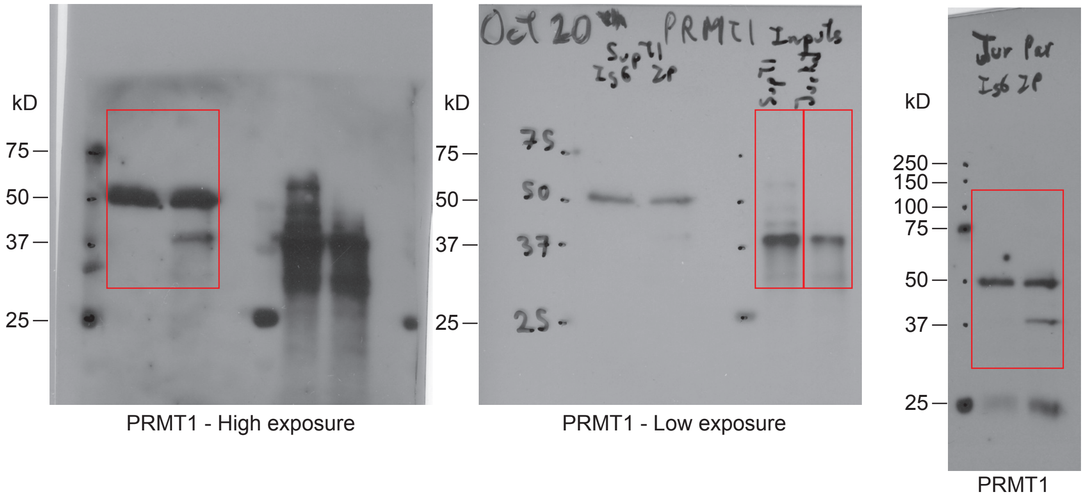

Supplementary Figure 13

Supplementary Figure 13

Uncropped images of blots shown in Figure 3 with molecular weight markers indicated.

Figure 4a

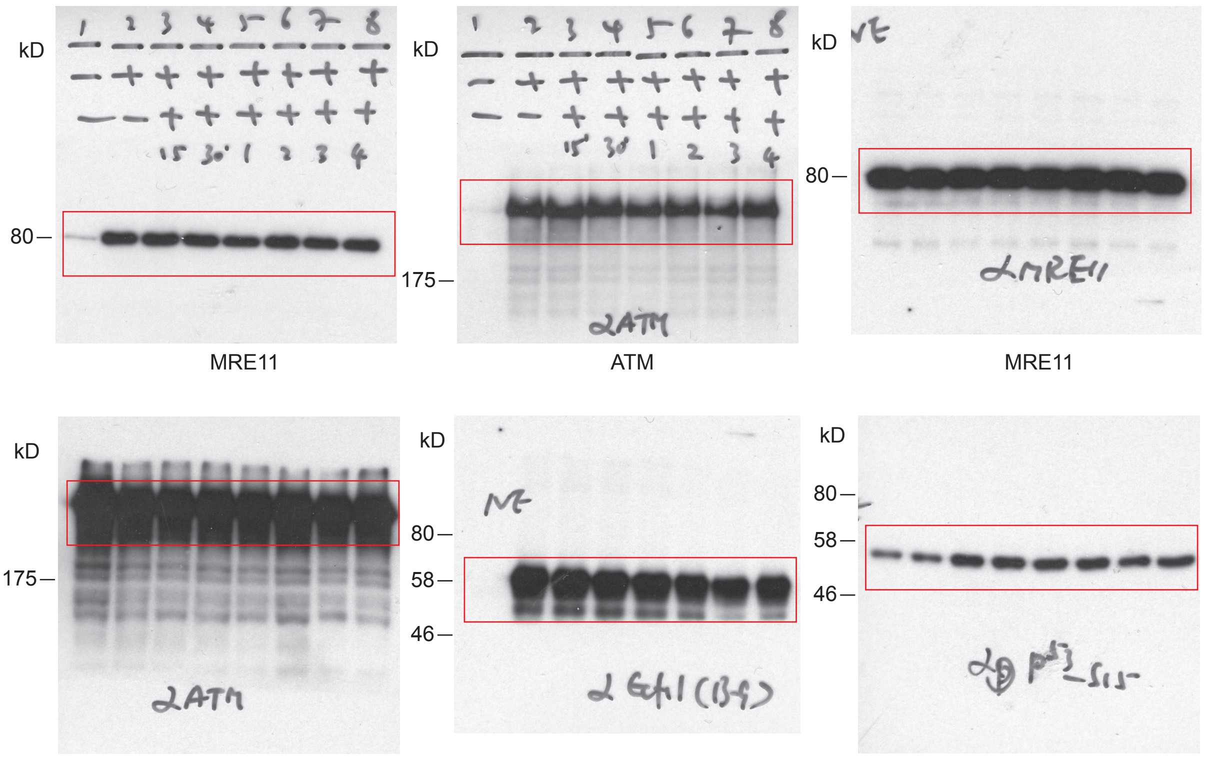

MRE11 ATM MRE11

ATM GFI1 p53 pS15

Supplementary Figure 14

Supplementary Figure 14
Uncropped images of blots shown in Figure 4 panel **a** with molecular weight markers indicated.

Figure 5a

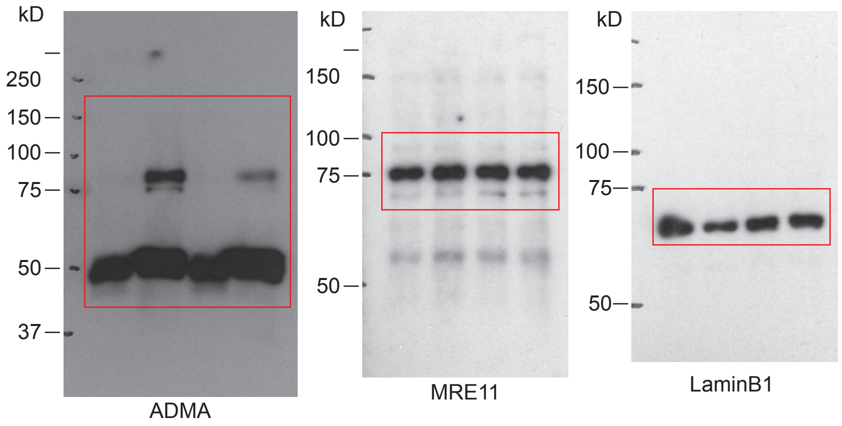

Figure 5b

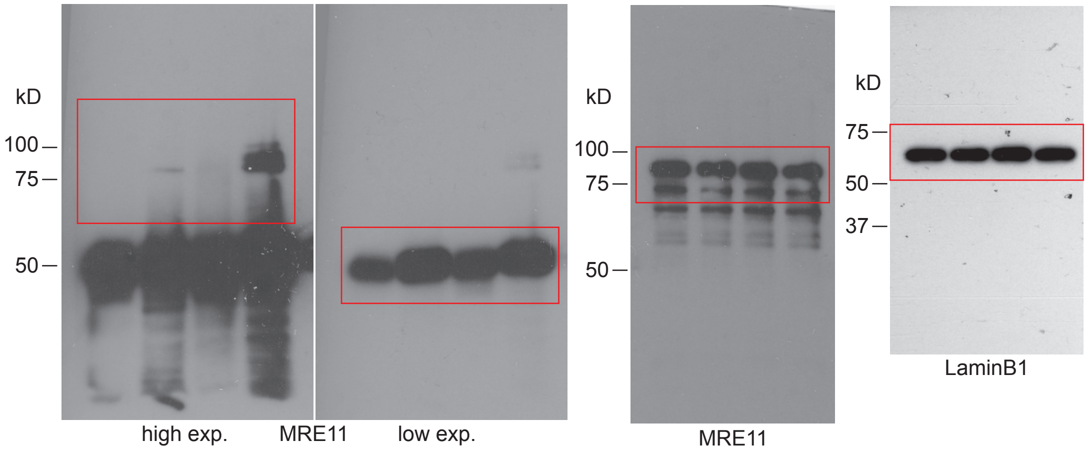

Figure 5c

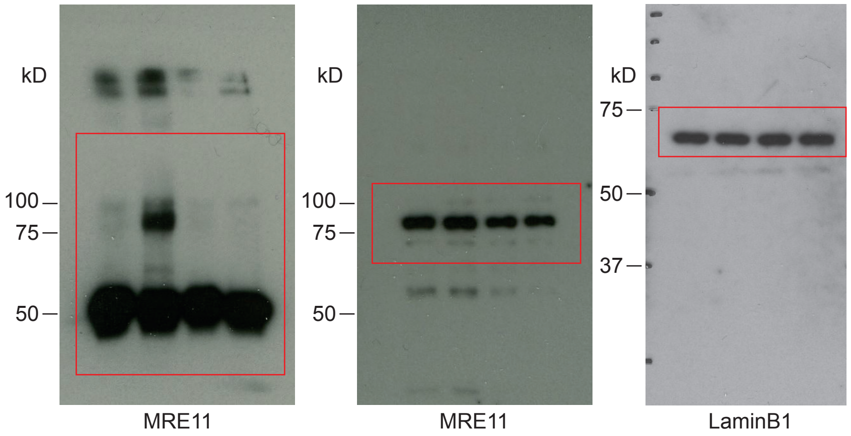

Figure 5d

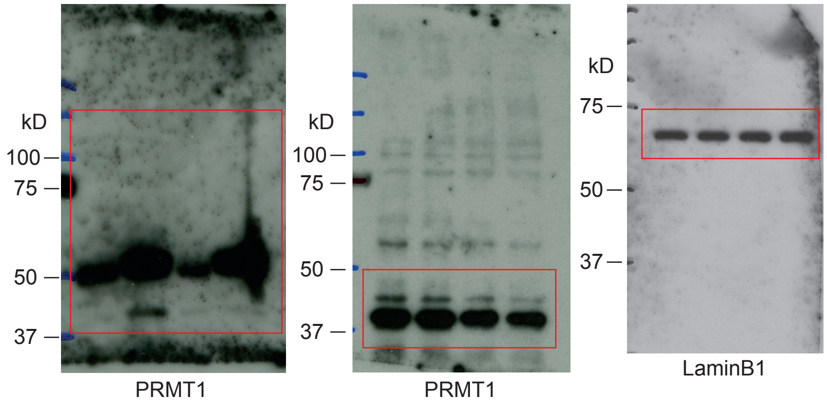

Supplementary Figure 15

Supplementary Figure 15

Uncropped images of blots shown in Figure 5 panels **a** through **d** with molecular weight markers indicated.

Figure 5e

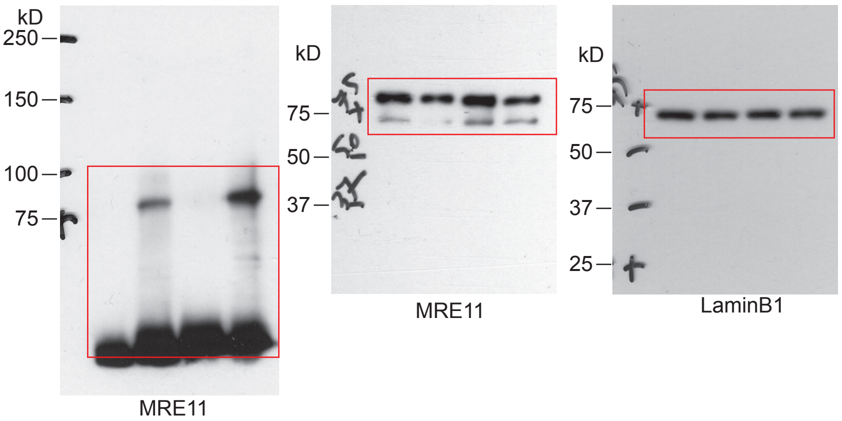

Figure 5f

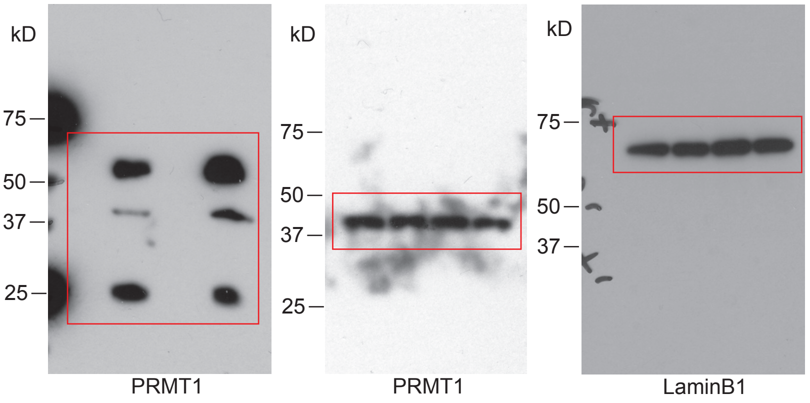

Figure 5g

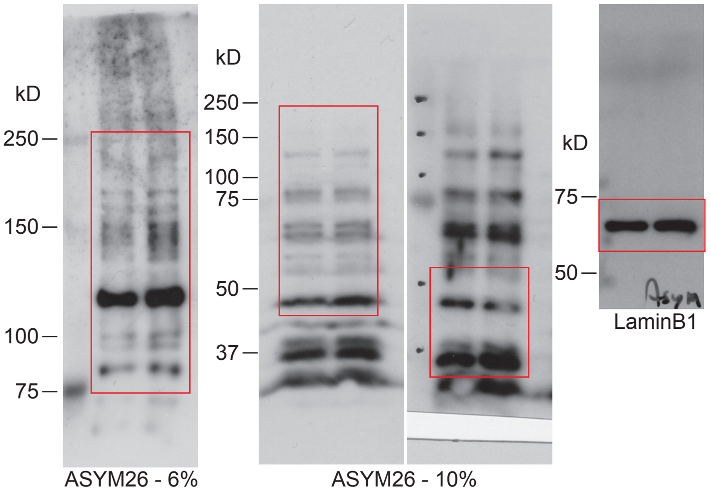

Figure 5h

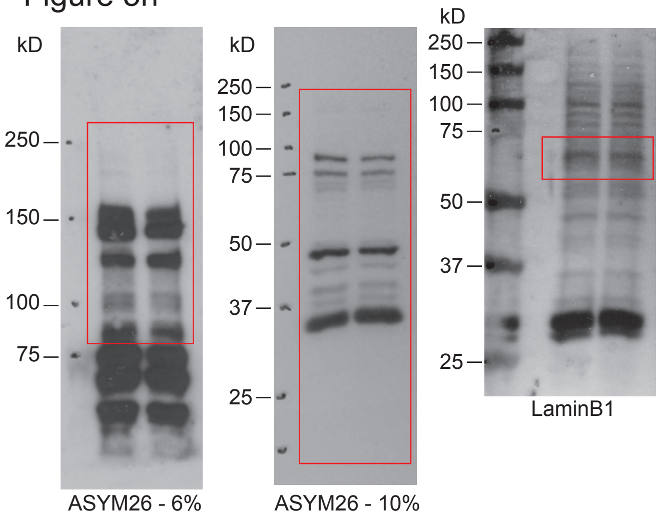

Supplementary Figure 16

Supplementary Figure 16

Uncropped images of blots shown in Figure 5 panels **e** through **h** with molecular weight markers indicated.

Figure 6a

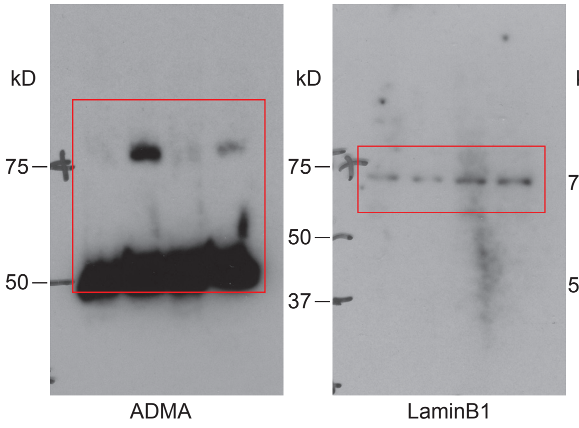

Figure 6c

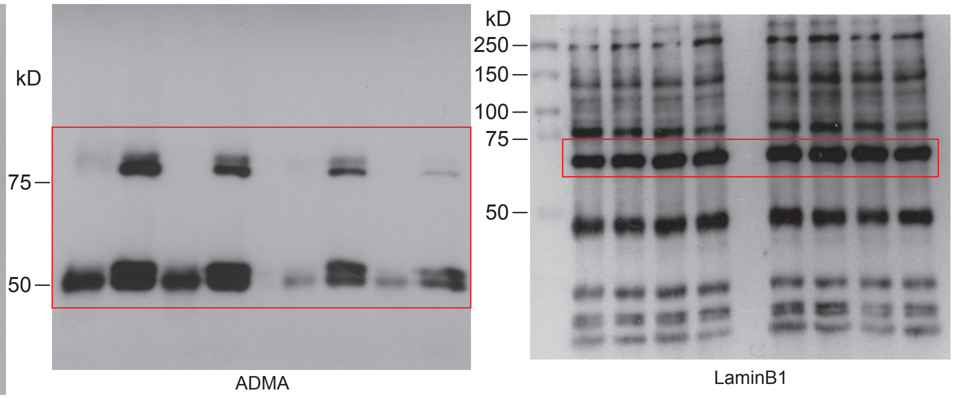

Supplementary Figure 1a, b & c

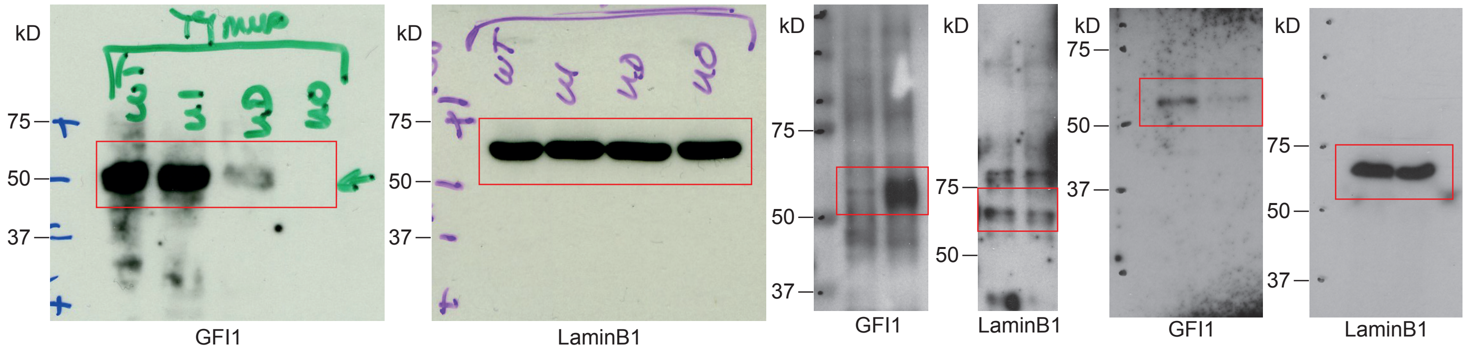

Supplementary Figure 4b

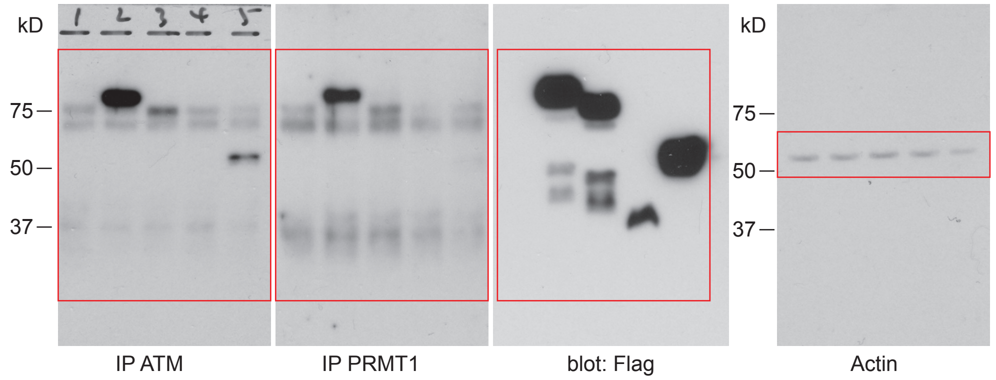

Supplementary Figure 4c

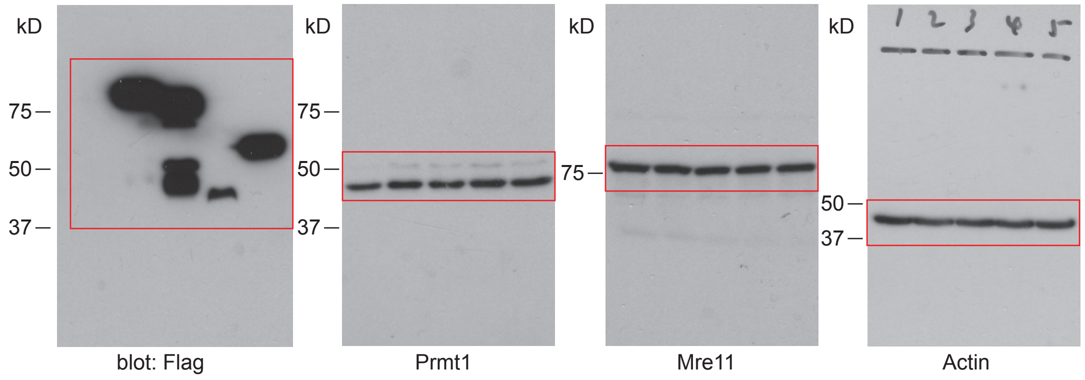

Supplementary Figure 17

Supplementary Figure 17
Uncropped images of blots shown in Figure 6 and Supplementary Figures 1 and 4 with molecular weight markers indicated.

Supplementary Figure 5a & b

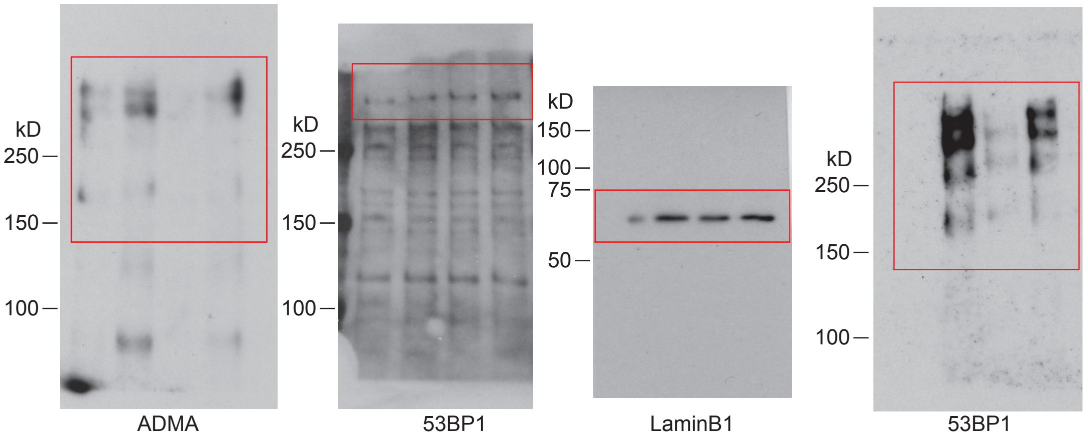

Supplementary Figure 5c

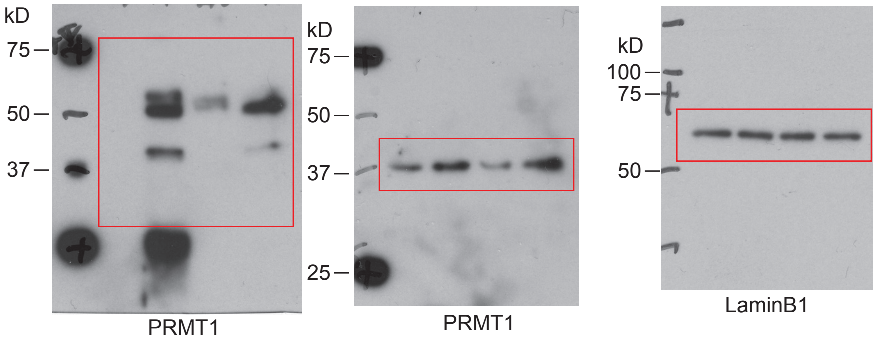

Supplementary Figure 5d

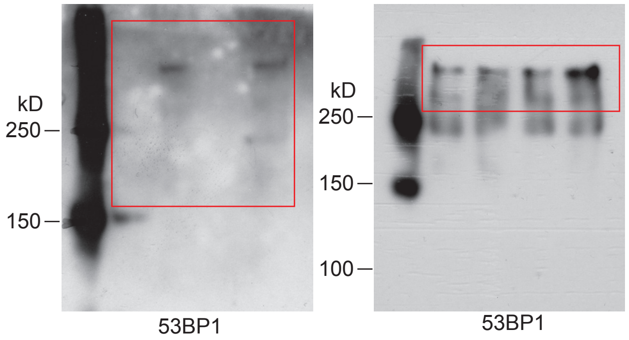

Supplementary Figure 18

Supplementary Figure 18
Uncropped images of blots shown in Supplementary Figure 5 with molecular weight markers indicated.

Supplementary Figure 7a

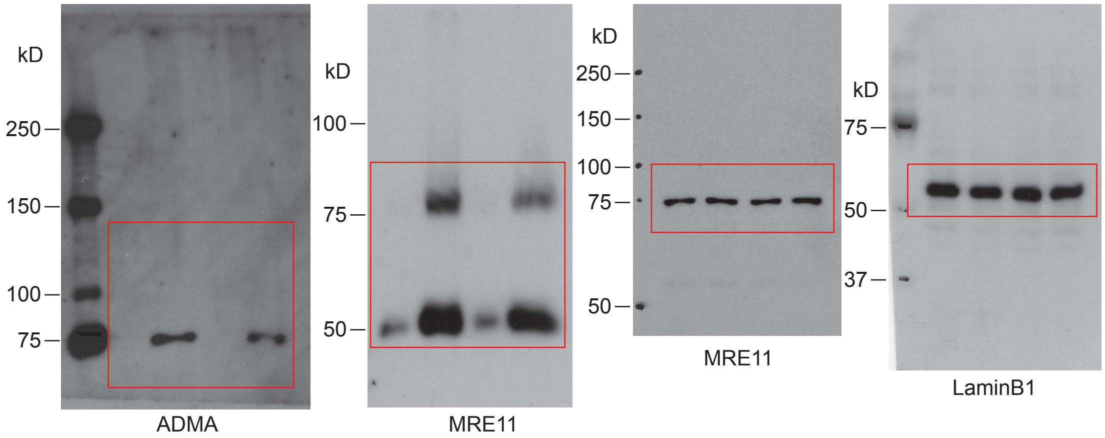

Supplementary Figure 7b

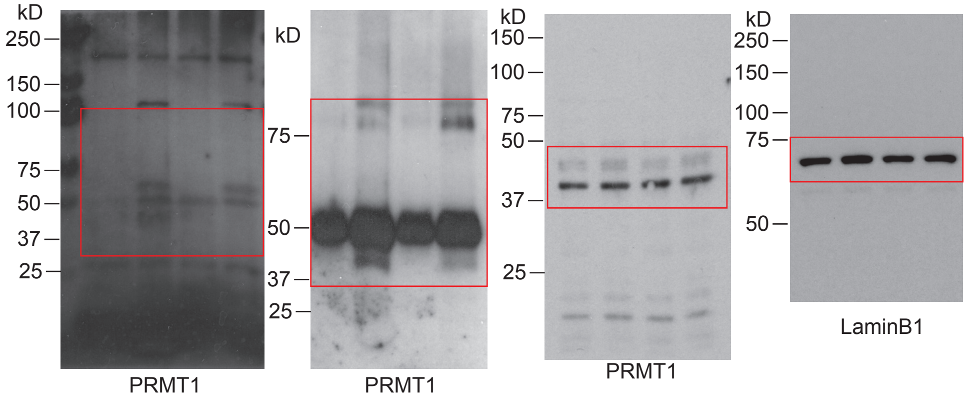

Supplementary Figure 7c

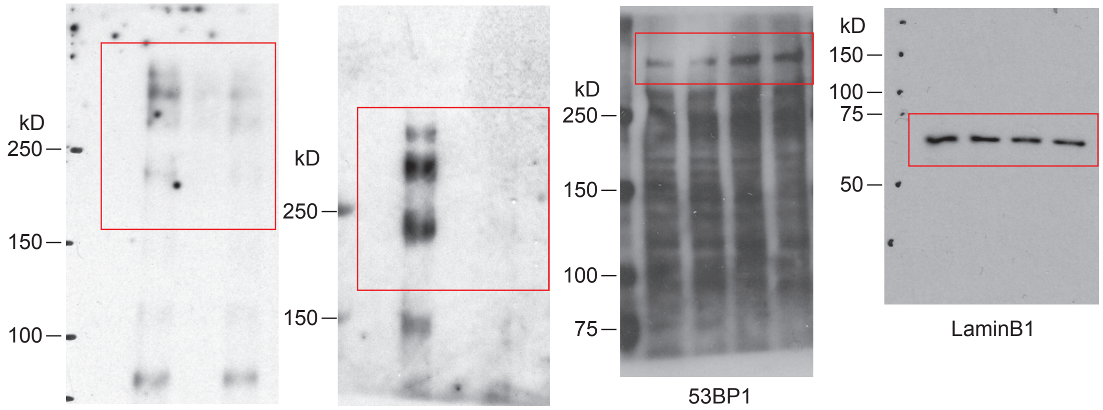

Supplementary Figure 7d

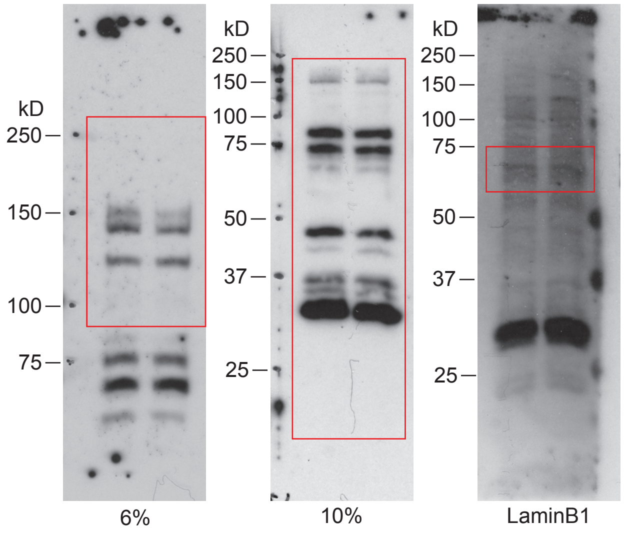

Supplementary Figure 19

Supplementary Figure 19

Uncropped images of blots shown in Supplementary Figure 7 with molecular weight markers indicated.

Supplementary Figure 8a

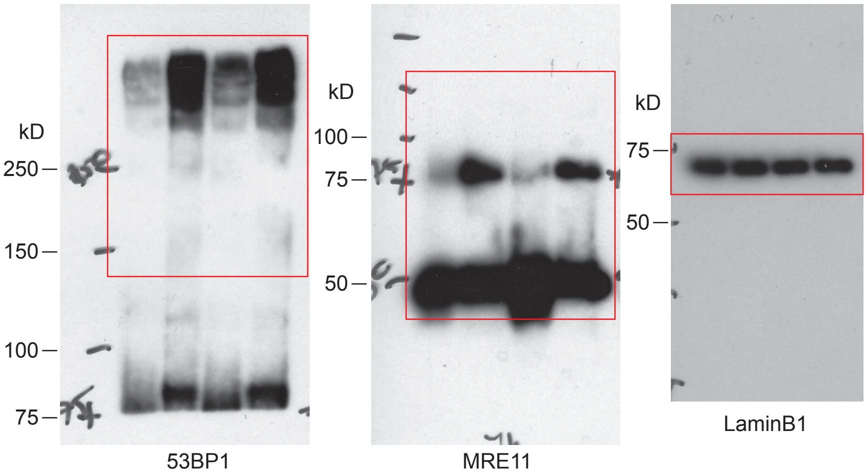

Supplementary Figure 8c

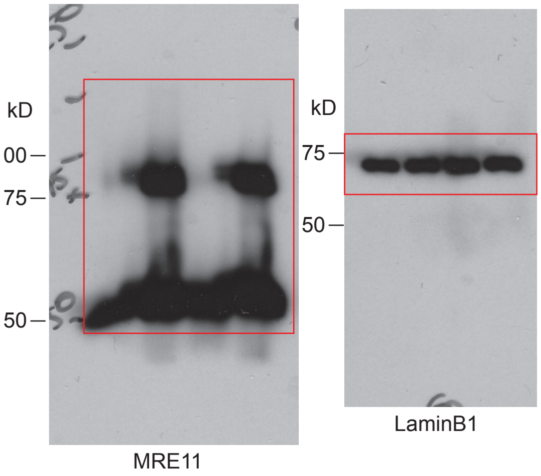

Supplementary Figure 8b

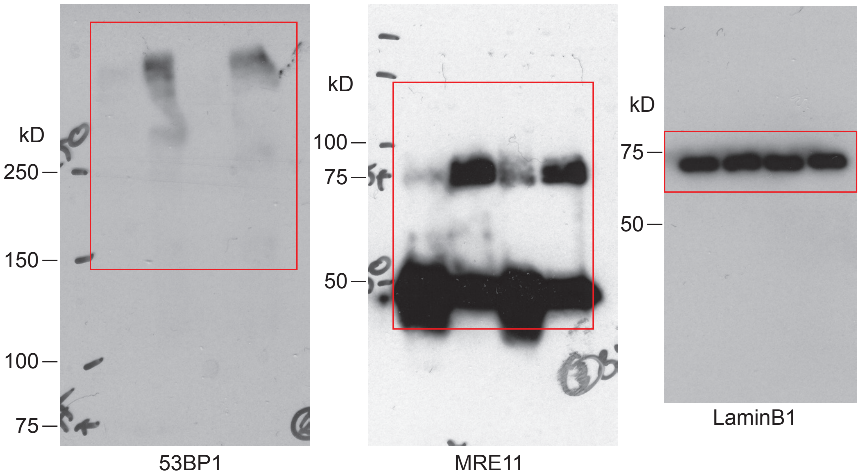

Supplementary Figure 8d

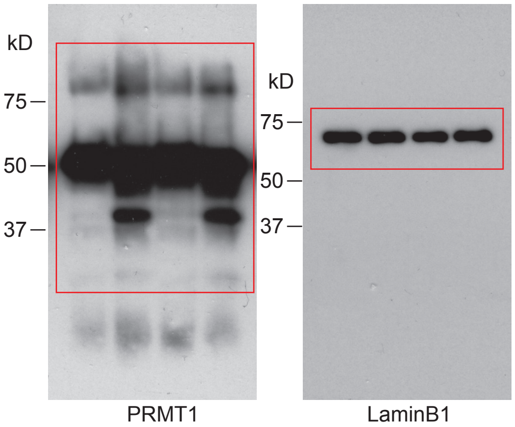

Supplementary Figure 8e

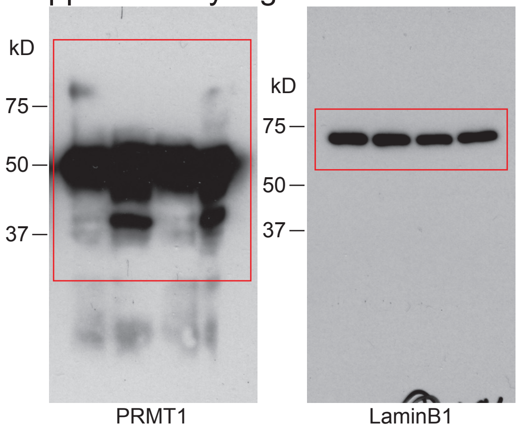

Supplementary Figure 8f

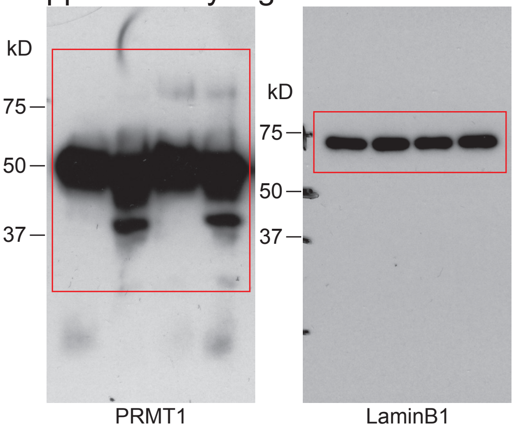

Supplementary Figure 20

Supplementary Figure 20
Uncropped images of blots shown in Supplementary Figure 8 with molecular weight markers indicated.

Supplementary Figure 9c & d

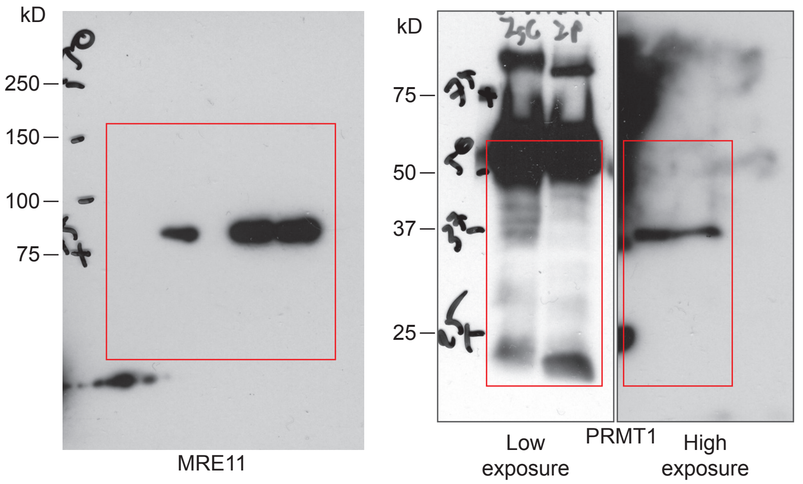

Suppl. Figure 10a

Suppl. Figure 10b

Suppl. Figure 10d

Suppl. Figure 10e

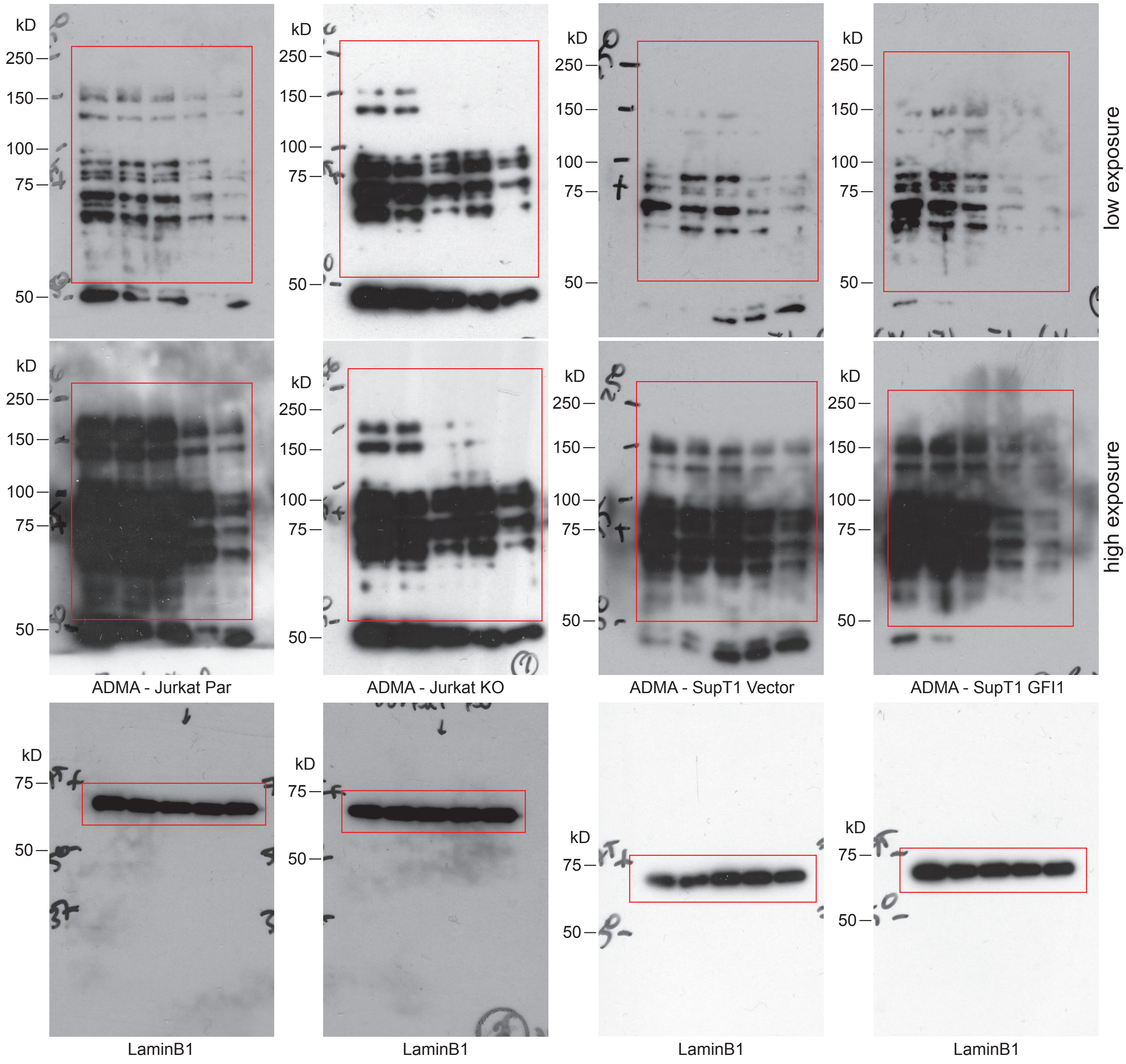

Supplementary Figure 21

Supplementary Figure 21
Uncropped images of blots shown in Supplementary Figures 9 & 10 with molecular weight markers indicated.
