# Supplementary material for: GFI1 facilitates efficient DNA repair by regulating PRMT1 dependent methylation of MRE11 and 53BP1
Source: Nat Commun. 2018 Apr 12;9:1418. doi: 10.1038/s41467-018-03817-5 (PMC5897347; doi:10.1038/s41467-018-03817-5)
Supplement: Supplementary file 3 — Description of Additional Supplementary Files [file 41467_2018_3817_MOESM3_ESM.pdf]

Description of Additional Supplementary Files

File Name: Supplementary Data 1
Description: Processed proteomics listing all GFI1 binding partners considered significant.

File Name: Supplementary Data 2
Description: List of sample sizes for all data points for each figure.
